# Significant Association Between Abundance of Gut Microbiota and Plasma Levels of microRNAs in Individuals with Metabolic Syndrome and Their Potential as Biomarkers for Metabolic Syndrome: A Pilot Study

**DOI:** 10.3390/genes16101161

**Published:** 2025-09-30

**Authors:** Sanghoo Lee, Jeonghoon Hong, Yiseul Kim, Hee-Ji Choi, Jinhee Park, Jihye Yun, Yun-Tae Kim, Kyeonghwan Choi, SaeYun Baik, Mi-Kyeong Lee, Kyoung-Ryul Lee

**Affiliations:** 1 Companion Biomarker Center, Seoul Clinical Laboratories Healthcare Inc., Yongin-si 16954, Gyeonggi-do, Republic of Korea; 2 Department of Diagnostic Test, Seoul Clinical Laboratories, Yongin-si 16954, Gyeonggi-do, Republic of Korea; 3 HANARO Medical Foundation, Seoul 03159, Republic of Korea; 4 Central Laboratory, Seoul Clinical Laboratories Healthcare Inc., Yongin-si 16954, Gyeonggi-do, Republic of Korea; 5 Division of Laboratory Medicine, Seoul Clinical Laboratories, Yongin-si 16954, Gyeonggi-do, Republic of Korea

**Keywords:** metabolic syndrome, gut microbiota, *Bacteroidetes*, *Firmicutes*, miR-122, miR-370, biomarker, NGS, RT-qPCR

## Abstract

**Background/Objectives:** The relationship between gut microbiota (GM) and microRNAs (miRs) related to lipid metabolism in individuals with metabolic syndrome (MetS) remains unclear. This pilot study examined the relationship between *Bacteroidetes* and *Firmicutes* abundance at the phylum level and the plasma levels of miR-122 and miR-370, both of which are associated with lipid metabolism, in Korean individuals with MetS and in healthy controls. We also evaluated the potential of these miRs as biomarkers for MetS. **Methods:** This study enrolled 7 individuals with MetS and 8 controls. The abundance of GM was analyzed by 16S rRNA amplicon sequencing. To evaluate the relationship between the dominant phyla in the 2 groups, the log ratio of *Firmicutes* to *Bacteroidetes* (F/B) was calculated using a centered log-ratio (CLR) transformation. The abundance of the 2 plasma miRs was also quantified by real-time quantitative PCR (RT-qPCR). Pearson’s and Spearman’s correlation analyses were then performed to evaluate the relationship between *Bacteroidetes* and *Firmicutes* abundance, the clinical parameters, and plasma levels of the 2 miRs. Additionally, the area under the curve (AUC) value of the receiver operating characteristic (ROC) curve was calculated to evaluate the potential of the 2 miRs as MetS biomarkers. **Results:** The 2 most abundant phyla were *Bacteroidetes* and *Firmicutes*. *Bacteroidetes* made up an average of 24.7% in the MetS group and 69.7% in the control group. Meanwhile, the average abundance of *Firmicutes* was 69.8% in the MetS group and 26.5% in the control group. The log F/B ratios in the MetS and control groups were 0.7 ± 0.5 and −0.4 ± 0.1 (*p* < 0.001), respectively. FDR analysis revealed significant correlations between *Bacteroidetes* abundance and BMI, DBP, FBG, total chol, insulin and HOMA-IR (FDR-adjusted *p* < 0.05), as well as between *Firmicutes* abundance and BMI, FBG, total chol, insulin and HOMA-IR (FDR-adjusted *p* < 0.05). Plasma levels of the 2 miRs differed significantly between the MetS and control groups: miR-122 (1.43 vs. 0.73; *p* = 0.0065) and miR-370 (1.39 vs. 0.83; *p* = 0.0089). The AUC values for miR-122 and miR-370 were 0.946 (*p* < 0.001) and 0.964 (*p* < 0.001), respectively. Pearson’s and Spearman’s correlation analyses revealed significant negative correlations between *Bacteroidetes* abundance and levels of miR-122 (*p* = 0.0048 and *p* = 0.0045, respectively) and miR-370 (*p* = 0.0003 and *p* < 0.0001, respectively), as well as significant positive correlations between *Firmicutes* abundance and levels of miR-122 (*p* = 0.0038 and *p* = 0.0027, respectively) and miR-370 (*p* = 0.0004 and *p* < 0.0001, respectively). However, as our exploratory findings were based on a small sample size, the high correlation results may partly reflect the separation between the MetS and control groups. **Conclusions:** Our exploratory findings suggest that the GM abundances of individuals with MetS may be significantly associated with plasma levels of miR-122 and miR-370, which are related to lipid metabolism. These miRs may therefore serve as potential MetS biomarkers.

## 1. Introduction

The prevalence of MetS is increasing worldwide. Notably, it is increasing rapidly in the United States, with the most significant rise observed among Asian and Hispanic adults compared to other racial and ethnic groups [1]. The prevalence of MetS is also rising significantly in East Asia [2]. Large-scale cohort studies have confirmed this trend in the Republic of Korea [3,4].

MetS is characterized by various risk factors that increase the likelihood of developing heart disease, chronic kidney disease, cancer, and type 2 diabetes (T2D) [5,6]. These risk factors are closely related to hypertension, high fasting glucose levels, abnormal blood lipid levels, and obesity. Abdominal obesity in particular can be attributed to genetic predisposition, gut microbiota, lifestyle, and dietary habits [7,8,9,10]. Therefore, it is important to detect individuals with MetS early and reduce its risk through lifestyle management and intervention.

The gut is the organ with the highest microbial population in the human body, estimated to be around 10^13^ or more [11,12]. Most of these microbes belong to the major phyla *Firmicutes* and *Bacteroidetes*, accounting for 60–80% and 20–30% of the total GM, respectively [13]. Dysbiosis, or an imbalance in the composition of gut microbes, is identified as one of the main causes of MetS [14,15]. Decreased GM diversity or increased levels of certain harmful bacteria can lead to impaired metabolic function, an increased inflammatory response and insulin resistance, thereby raising the risk of developing MetS [16,17].

Many studies have reported that people with MetS or its risk factors have an elevated F/B ratio, resulting from higher *Firmicutes* and lower *Bacteroidetes* abundances. This suggests that the F/B ratio plays an important role in MetS [18,19,20,21,22].

MicroRNAs (miRs) are among the most abundant gene regulators in humans. These small (~22 nucleotides), non-coding, single-stranded RNA molecules are known to play a crucial role in negatively regulating gene expression at the post-transcriptional level [23,24]. Under pathogenic regulatory conditions, miRs play an important role in the pathogenesis of diseases, such as T2D and cardiovascular diseases (CVDs). Studies have reported on the importance of circulating miR expression profiles and functions in regulating multiple diseases, suggesting that they could be potential biomarkers for diagnosing human diseases [25,26,27]. Due to their significant regulatory roles in MetS pathogenesis, miRs have great potential as early biomarkers and therapeutic targets for MetS, a risk factor for developing T2D and CVD. Previous studies have demonstrated that various miRs, including miR-33a/b, miR-122, and miR-370, play a significant role in lipid metabolism [28]. In particular, several key genes involved in fatty acid synthesis and oxidation are regulated by miR-122. Furthermore, it has been demonstrated that miR-370 regulates the expression of miR-122 and downregulates the expression of genes that regulate fatty acid oxidation [29]. Additionally, a recent study has reported that miR-15a-5p and miR-17-5p could act as potential MetS biomarkers [30].

GM and miRs, which are known to interact with gut epithelial cells, influence various signaling pathways and regulate genetic transcription, acting as crucial regulatory factors for human health [31,32,33]. Disruption to the bidirectional interaction between GM and miR could also influence the development of various chronic diseases, including MetS, T2D, autoimmune diseases, and cancers [34,35]. Therefore, improving our understanding of the underlying mechanisms of these bidirectional interactions could help to establish novel therapeutic targets for treating MetS and related metabolic diseases.

Although recent studies have examined the interaction between the GM and miRs, the relationship between GM profiles and miR-122 and miR-370, both of which are both related to lipid metabolism, has not yet been reported in individuals with MetS. Our exploratory pilot study reveals a significant correlation between plasma levels of miR-122 and miR-370 and GM abundance in Korean individuals with MetS. Through AUC-ROC curve analysis, we also demonstrated that these 2 miRs may have potential as MetS biomarkers.

Participants who completed a dedicated questionnaire were classified by a physician as having MetS or as healthy controls, based on comprehensive blood test results; information about their lifestyles, diets, medications, and other metabolic diseases; and other details provided in the questionnaire. Participants who were taking two or more combination medications, undergoing chemotherapy, or taking anticancer drugs were excluded from the study.

## 2. Materials and Methods

### 2.1. Study Participants

A total of 48 study participants were initially enrolled in our MetS cohort study between September 2018 and August 2019. However, only 7 MetS and 8 healthy controls ultimately participated in the study, based on strict classification criteria (Table 1). Participants were classified as having MetS or as healthy controls based on a physician’s diagnosis, informed by the results of comprehensive blood tests and a questionnaire detailing information about participants’ lifestyles, diets, medications, and other metabolic diseases. All of the study participants completed the questionnaire. Participants taking two or more combination medications, undergoing chemotherapy, or taking anticancer drugs were excluded from the study. All blood tests, questionnaire completion, and comprehensive health check-ups were conducted for the participants at the HANARO Medical Foundation in Seoul, Republic of Korea.

Individuals with MetS were diagnosed and enrolled according to the harmonized definition of the International Diabetes Federation/National Heart, Lung, and Blood Institute/American Heart Association/International Association for the Study of Obesity [5]. The MetS group had 3 or more of the following clinical characteristics: hypertension (systolic pressure ≥ 130 mmHg and/or diastolic pressure ≥ 85 mmHg), hyperglycemia (fasting glucose: ≥100 mg/dL), increased triglycerides (TGs) (≥150 mg/dL), and decreased HDL-cholesterol (<40 mg/dL in men and <50 mg/dL in women). Abdominal obesity was defined according to the definition of the Korean Academy of Family Medicine [36], using waist circumference as the criterion: >90 cm for men and >85 cm for women.

All study participants provided written informed consent. The study protocol (SCL IRB-2018-31) was approved by the Institutional Review Board of Seoul Clinical Laboratories.

### 2.2. Collection of Fecal and Plasma Samples from Study Participants

A total of 15 fresh stool samples were collected for the GM profile from 7 subjects with MetS and 8 controls. The samples were collected using sterile, DNase-free containers dedicated to this purpose and stored at −80 °C until DNA extraction. Whole blood samples were also collected from each study participant, separated by immediate centrifugation at 3000× *g* for 15 min at 4 °C, and plasma was recovered. The plasma samples were then stored at −80 °C until the analysis of miRs.

### 2.3. Bacterial DNA Extraction, 16S Metagenomic Sequencing Library Preparation, and Next-Generation Sequencing (NGS)

Genomic DNA was extracted from stool samples using the PowerFecal^®^ DNA Isolation Kit (MO BIO Laboratories Inc., Carlsbad, CA, USA), following the manufacturer’s instructions. The concentration and quality of the stool DNA were checked using a NanoPhotometer N60 UV/visible spectrophotometer (Implen GmbH, Munich, Germany). The DNA samples were stored at −20 °C until the GM analysis. Sequencing libraries targeting the hypervariable V3–V4 region of the 16S rRNA gene were prepared using the Illumina 16S metagenomics sequencing library preparation workflow. This is a two-step PCR workflow that amplifies the V3 and V4 regions of the 16S gene in a single amplicon. The primer sequences targeting these regions are as follows: the forward (50 bp) = 5′-TCGTCGGCAGCGTCAGATGTGTATAAGAGACAGCCTACGGGNGGCWGCAG-3′ and the reverse (55 bp) was 5′-GTCTCGTGGGCTCGGAGATGTGTATAAGAGACAGGACTACHVGGGTATCTAATCC-3′. Seven samples from MetS and 8 control samples were prepared using the Illumina 16S NGS library preparation protocol and the Nextera^®^ XT DNA Index Kit. The PCR products were purified using AMPure XP beads (Thermo Fisher Scientific Inc., Waltham, MA, USA) and their size was confirmed using a TapeStation4200 (Agilent Technologies Inc., Santa Clara, CA, USA). The NGS libraries were then pooled at an equal concentration. Six hundred cycles of 2 × 300 bp paired-end reads were produced on the Illumina MiSeqDx NGS system using the MiSeq Reagent Kit v3 (Illumina, San Diego, CA, USA). The resulting sequence reads were distributed equally across the samples, revealing uniform coverage.

### 2.4. 16s rRNA Amplicon Sequence Analysis

To determine the 16S metagenomic differences between the MetS and the control groups, amplicon sequence variants (ASVs) were calculated [37]. To ensure the quality control of the raw data, trimmomatic (v0.39) was used to trim portions of the reads with base quality scores of 15 or less (phred scale) from the front and back of [38]. All parts with an average base quality score of 15 or less were trimmed using a sliding window of 4 bp, and the reads with a total length of 250 bp or less were discarded. ASVs were produced using filtered reads by DADA2 [37]. The default settings were used for all ASV calling steps.

### 2.5. Classification of Amplicon Sequences According to Taxonomy

We performed a taxonomic classification of the ASVs produced for quantification, classifying them at the genus level. This was achieved using the ‘assignTaxonomy’ function in DADA2 and the SILVA database (v138) [39]. We created a feature table using the ASV’s taxonomic and abundance information, calculated their abundances [40]. Taxonomy, abundance, and clinical information were integrated and analyzed using the phyloseq package, and compositional analysis was performed using the microbiome package [41,42,43]. To process zeros, we added half of the minimum read count to all zeros. Additionally, we performed a centered log-ratio transformation on feature abundance for further statistical analysis [42].

### 2.6. RNA Extraction from Blood Sample and Quantification of Plasma miRs by RT-qPCR

Total RNA was isolated from 400 μL of plasma using a mirVana^TM^ PARIS^TM^ RNA kit (Life Technologies Inc., Carlsbad, CA, USA) following the manufacturer’s instructions with modifications. To normalize variation between samples, 5 pmol of synthetic *Caenorhabditis elegans* miR-cel-39-3p (Qiagen Inc., Hilden, Germany) was added to each sample after the addition of 2× Denaturing Solution (Ambion Inc., Austin, TX, USA). The RNA was dissolved in 100 μL of RNase-free water, and then stored at −80 °C until analysis. Total RNA was reverse transcribed using a Taq-Man Advanced MicroRNA cDNA Synthesis Kit (Thermo Fisher Scientific Inc., USA), according to the manufacturer’s instructions. To quantify the miR in the plasma of each study participant by RT-qPCR, 5 μL of the diluted cDNA product was used as a template in a 20 μL reaction containing 1 μL of TaqMan Advanced miRNA Assay (Thermo Fisher Scientific Inc., USA), 10 μL of TaqMan 2× Fast Advanced Master Mix (Thermo Fisher Scientific Inc., USA), and 4 μL of RNase-free water. qRT-PCR was performed using a 7500 Fast Real-Time PCR System (Applied Biosystems Inc., Foster City, CA, USA) with the following program: 95 °C for 20 s, followed by 40 cycles of 95 °C for 3 s and 60 °C for 30 s. Data were analyzed using the automatic Ct setting to assign the baseline and determine the threshold. The relative expression level of each individual miR, normalized to cel-miR-39, was calculated using the 2^−ΔΔCt^ method [44].

### 2.7. Statistical Analysis

The results are expressed as the mean ± standard deviation (SD). Statistical analyses were performed using MedCalc^®^ software (v23.2.8). Differences in clinical parameters between the MetS and control groups were evaluated using the Wilcoxon–Mann–Whitney test. Differences in GM abundance between the two groups at the phylum and genus levels were assessed using the Mann–Whitney U test. Multiple testing was adjusted using a Benjamini–Hochberg FDR-adjusted *p*-value calculation. The abundance values of the two dominant phyla, *Firmicutes* and *Bacteroidetes*, in each group were normalized using the declared CLR transformation, and the results were presented as log F/B ratios. Associations between the abundance of the *Bacteroidetes* and *Firmicutes* phyla, the clinical parameters of the study participants and the relative expression levels of the 2 miRs were evaluated using Pearson’s and Spearman’s correlation analyses. The AUC value of ROC curve was calculated to evaluate the potential of the 2 miRs as MetS biomarkers using the MedCalc^®^ software (v23.2.8). *p* < 0.05 was considered statistically significant.

We also calculated the power of our pilot study using both the sample size calculator and the post hoc power calculator provided by ClinCalc (https://clincalc.com/).

## 3. Results

### 3.1. Clinical Characteristics of Participants with MetS and Controls

The clinical characteristics of 8 controls and 7 individuals with MetS are presented in Table 1. Analysis of the clinical parameters of all participants in the study revealed statistically significant differences between the MetS and control groups (*p* < 0.05) for weight, WC, BMI, SBP, DBP, FBG, total chol, AST, ALT, γ-GTP, hs-CRP, insulin, and HOMA-IR, but not for age, height, HDL chol, LDL chol, TG, serum Cr, and eGFR. Weight, WC, BMI, FBG, γ-GTP, hs-CRP, insulin, and HOMA-IR exhibited highly significant differences between the 2 groups (*p* < 0.003).

### 3.2. Comparison of GM Composition Between MetS and Control Groups at the Phylum and Genus Levels

**Figure 1:**
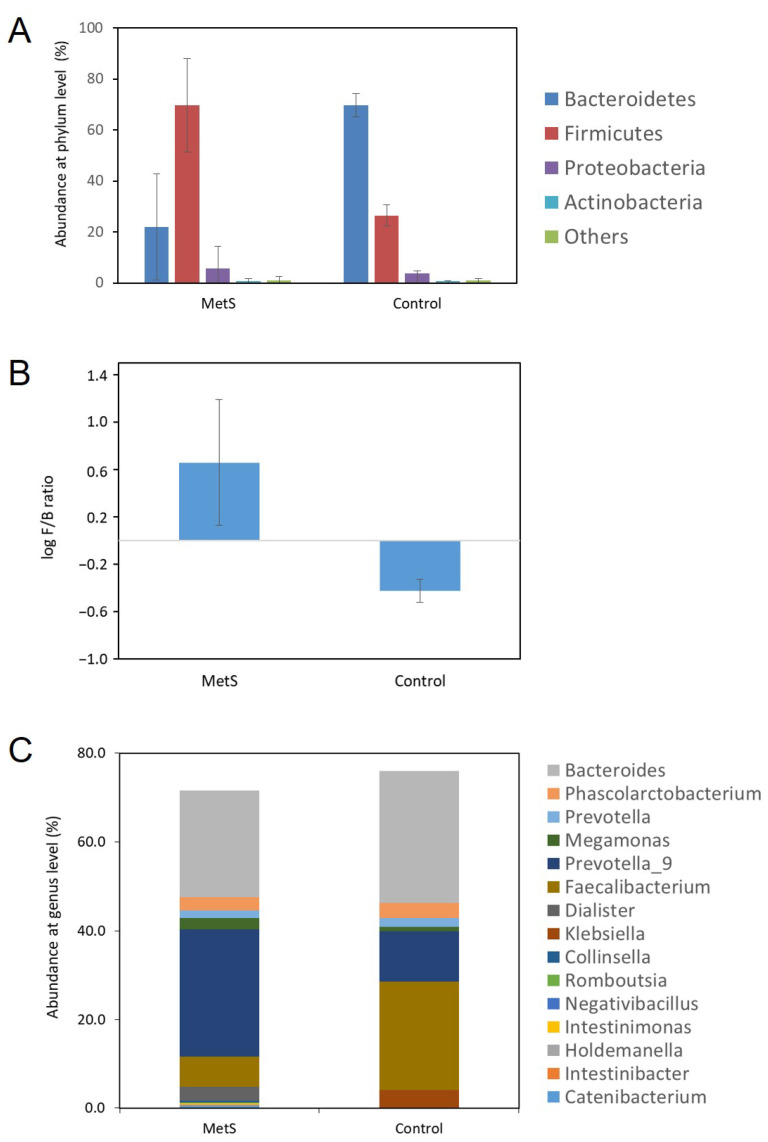
Characteristics of the GM between the MetS and control groups at the phylum and genus levels. (**A**) Relative abundances at the phylum level between the two groups. (**B**) The log F/B ratio in the two groups. (**C**) The 15 most abundant genera were detected in both groups. Specifically, *Prevotella_9* was identified as one of the dominant genera in both groups, with a relative abundance of ≥5%. However, no statistical significance was observed between the two groups (*p* = 0.1232).

Our pilot study revealed that the GM of both the MetS and control groups was dominated by the phyla *Bacteroidetes* and *Firmicutes* (Figure 1). The data showed that the relative abundance of the phylum *Firmicutes* in individuals with MetS was significantly higher than in the control group (average 69.7% vs. 26.5% [95% CI −60.0, −26.3], *p* = 0.0005), whereas the relative abundance of *Bacteroidetes* in the control group was significantly higher than in individuals with MetS (average 69.8% vs. 24.7% [95% CI 27.2, 62.8], *p* = 0.0006) (Figure 1A). Furthermore, the log F/B ratio revealed a statistically significant difference between the MetS and control groups (0.7 ± 0.5 [95% CI 0.1587, 1.1490] vs. −0.4 ± 0.1 [95% CI −0.5009, −0.3645], *p* < 0.001) (Figure 1B). However, no significant differences were observed in the relative abundance of the *Proteobacteria* phylum (average 5.5% vs. 3.7% [95% CI −10.0, 6.4], *p* = 0.6072) or the *Actinobacteria* phyla (average 5.5% vs. 3.7% [95% CI −0.9, 0.6], *p* = 0.7609) between the MetS and control groups (Figure 1A).

At the genus level, the GM characteristics of the MetS and control groups were analyzed using the Mann–Whitney U test. A total of 116 genera were identified (Supplementary Table S1). The 6 most abundant genera detected in the MetS group were *Prevotella_9* (28.74%), *Bacteroides* (23.92%), *Faecalibacterium* (6.79%), *Dialister* (3.11%), *Phascolarctobacterium* (3.03%), and *Megamonas* (2.53%), whereas in the control group they were *Bacteroides* (29.83%), *Faecalibacterium* (24.51%), *Prevotella_9* (11.28%), *Klebsiella* (3.93%), *Phascolarctobacterium* (3.36%), and *Prevotella* (2.04%). The dominant genera with a relative abundance of ≥5% in both groups were therefore *Bacteroides*, *Prevotella_9*, and *Faecalibacterium* (Figure 1C). Eight genera showed a statistically significant difference between the 2 groups (*p* < 0.05, Supplementary Table S1). These were *Catenibacterium* (0.58%), *Intestinibacter* (0.13%), *Holdemanella* (0.17%), *Intestinimonas* (0.15%), *Negativibacillus* (0.03%), *Romboutsia* (0.11%), and *Collinsella* (0.46%) in the MetS group. In contrast, the only genus detected in the control group was *Klebsiella* (3.93%). However, FDR-adjusted *p*-values revealed no statistically significant differences between the 2 groups. This suggests that the findings of our pilot study are exploratory rather than definitive, given that the study was conducted with a relatively small sample size.

### 3.3. Association of GM with Clinical Parameters of the Study Participants at the Phylum Level

Pearson’s and Spearman’s correlation analyses were performed to examine the relationship between the abundance of the *Bacteroidetes* and *Firmicutes* phyla and the clinical parameters. A limited-scope FDR was also applied to pre-specified key traits (Table 2).

Pearson’s correlation analysis revealed significant negative correlations between WC (*r* = −0.7186, *p* = 0.0025), BMI (*r* = −0.6775, *p* = 0.0055), SBP (*r* = −0.5686, *p* = 0.0270), DBP (*r* = −0.6300, *p* = 0.0118), FBG (*r* = −0.6692, *p* = 0.0064), total chol (*r* = −0.6285, *p* = 0.0121), insulin (*r* = −0.6433, *p* = 0.0097), and HOMA-IR (*r* = −0.6635, *p* = 0.0070) and *Bacteroidetes* abundance. A FDR analysis of Pearson’s correlation results revealed statistical significance between the *Bacteroidetes* abundance and WC (*p* = 0.0425), BMI (*p* = 0.0468), DBP (*p* = 0.0334), FBG (*p* = 0.0363), total chol (*p* = 0.0294), insulin (*p* = 0.0330), and HOMA-IR (*p* = 0.0298). However, no statistical significance was found with SBP (*p* = 0.0574). Spearman’s correlation analysis revealed significant negative correlations between WC (*r_s_* = −0.6598, *p* = 0.0074), BMI (*r_s_* = −0.6897, *p* = 0.0044), SBP (*r_s_* = −0.6152, *p* = 0.0146), DBP (*r_s_* = −0.7460, *p* = 0.0014), FBG (*r_s_* = −0.7178, *p* = 0.0026), total chol (*r_s_* = −0.7751, *p* = 0.0007), LDL chol (*r_s_* = −0.5565, *p* = 0.0312), γ-GTP (*r_s_* = −0.6962, *p* = 0.0039), eGFR (*r_s_* = −0.5260, *p* = 0.0440), hs-CRP (*r_s_* = −0.6550, *p* = 0.0080), insulin (*r_s_* = −0.6971, *p* = 0.0039) and HOMA-IR (*r_s_* = −0.7168, *p* = 0.0026), and abundance of *Bacteroidetes*. FDR analysis of the Spearman’s correlation results also showed statistical significance between the *Bacteroidetes* abundance and BMI (*p* = 0.0377), DBP (*p* = 0.0060), FBG (*p* = 0.0088), total chol (*p* = 0.0019), γ-GTP (*p* = 0.0056), hs-CRP (*p* = 0.0091), insulin (*p* = 0.0041), and HOMA-IR (*p* = 0.0026). However, no statistical significance was found for WC (*p* = 0.1266), SBP (*p* = 0.0574), LDL chol (*p* = 0.0663), and eGFR (*p* = 0.0534) (Table 2).

Meanwhile, Pearson’s correlation analysis revealed significant positive correlations between WC (*r* = 0.793, *p* = 0.0004), BMI (*r* = 0.7765, *p* = 0.0007), DBP (*r* = 0.5637, *p* = 0.0286), FBG (*r* = 0.7705, *p* = 0.0080), total chol (*r* = 0.7884, *p* = 0.0005), insulin (*r* = 0.6907, *p* = 0.0044), and HOMA-IR (*r* = 0.7330, *p* = 0.0019) and *Firmicutes* abundance. Interestingly, there was also a significant negative correlation between HDL chol (*r* = −0.6368, *p* = 0.0107) and *Firmicutes* abundance. FDR analysis of the results of the Pearson’s correlation test revealed statistical significance between *Firmicutes* abundance and WC (*p* = 0.0068), BMI (*p* = 0.0040), FBG (*p* = 0.0227), total chol (*p* = 0.0043), HDL chol (*p* = 0.0260), insulin (*p* = 0.0150) and HOMA-IR (*p* = 0.0081), but not with DBP (*p* = 0.0608). Spearman’s correlation analysis revealed significant positive correlations between WC (*r_s_* = 0.6799, *p* = 0.0053), BMI (*r_s_* = 0.7107, *p* = 0.0030), SBP (*r_s_* = 0.6308, *p* = 0.0117), DBP (*r_s_* = 0.6976, *p* = 0.0038), FBG (*r_s_* = 0.6847, *p* = 0.0049), total chol (*r_s_* = 0.6893, *p* = 0.0045), γ-GTP (*r_s_* = 0.6643, *p* = 0.0069), hs-CRP (*r_s_* = 0.6625, *p* = 0.0071), insulin (*r_s_* = 0.7386, *p* = 0.0017), and HOMA-IR (*r_s_* = 0.7460, *p* = 0.0014), and *Firmicutes* abundance. FDR analysis of the Spearman’s correlation results also revealed statistical significance between *Firmicutes* abundance and BMI (*p* = 0.0253), DBP (*p* = 0.0163), FBG (*p* = 0.0165), total chol (*p* = 0.0127), γ-GTP (*p* = 0.0098), hs-CRP (*p* = 0.0081), insulin (*p* = 0.0018) and HOMA-IR (*p* = 0.0014). However, no statistical significance was found with WC (*p* = 0.0900) or SBP (*p* = 0.0662) (Table 2).

### 3.4. Expression Levels of the 2 miRs Related to Lipid Metabolism in MetS and Control Groups and Their Potential as MetS Biomarkers

**Figure 2:**
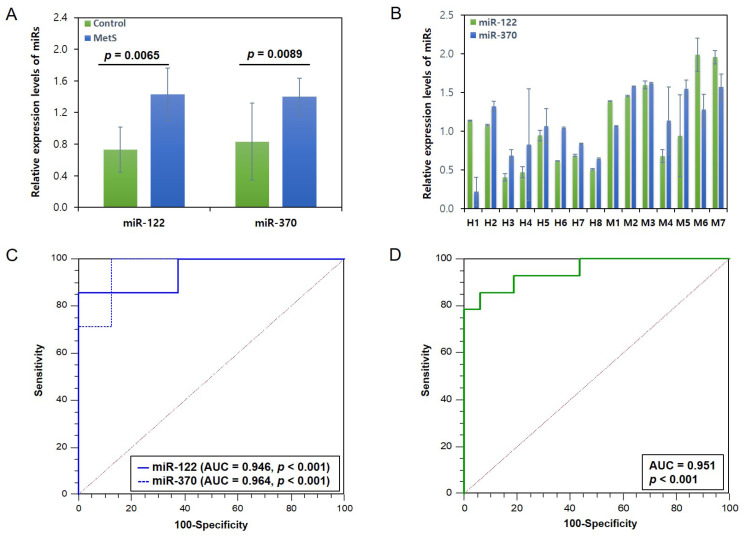
(**A**) Relative expression levels of miR-122 and miR-370 in the MetS and control groups. (**B**) Boxplots with individual data points for the miR panels. ROC curves for (**C**) miR-122 (bootstrap 95% CI, 0.804–0.997) and miR-370 (bootstrap 95% CI, 0.725–1.000), and (**D**) the 2 miRs combined (bootstrap 95% CI, 0.857–1.000).

The expression levels of miR-122 and miR-370 differed significantly between the plasma of the MetS and control groups. Notably, their expression levels were significantly higher in the MetS group than in the control group. The relative expression levels of miR-122 were 1.43 ± 0.48 and 0.73 ± 0.28 in the MetS and control groups, respectively (*p* = 0.0065), and those of miR-370 were 1.39 ± 0.23 and 0.83 ± 0.33 (*p* = 0.0089) (Figure 2A). Figure 2B shows boxplots with individual data points for miR-122 and miR-370 for each individual. Supplementary Table S2 presents the relative expression levels of the 2 miRs for each individual.

An ROC curve analysis was subsequently performed based on the relative expression levels of the 2 miRs to assess their ability to distinguish MetS from the control group. ROC curves were plotted for each of the 2 miRs, and their respective AUC values were calculated. These were 0.946 (bootstrap 95% CI 0.804–0.997, *p* < 0.001) for miR-122 and 0.964 (bootstrap 95% CI 0.725–1.000, *p* < 0.001) for miR-370 (Figure 2C). Combining the 2 miRs produced an AUC value of 0.951 (bootstrap 95% CI 0.857–1.000, *p* < 0.001) (Figure 2D), suggesting that using both miR-122 and miR-370 together did not significantly improve the AUC value compared to using them separately. As the ROC curve analysis was performed with a small sample size, the AUC values are exploratory-level results.

### 3.5. Correlation Between the Expression Levels of 2 miRs and the GM Abundances in the Individuals with MetS and Controls

To strengthen the reliability of our findings on phylum–miR associations, which were based on a small sample size, we conducted concurrent Spearman’s and Pearson’s correlation analyses.

Pearson’s correlation analysis revealed a significant negative correlation between levels of miR-122 and abundance of the *Bacteroidetes* phylum (*r* = −0.6890, *p* = 0.0048), as well as a significant positive correlation between levels of miR-122 levels and abundance of the *Firmicutes* phylum (*r* = 0.6980, *p* = 0.0038). Spearman’s correlation analysis also produced similar results, revealing a significant negative correlation between miR-122 levels and *Bacteroidetes* abundance (*r_s_* = −0.6890, *p* = 0.0045) and a significant positive correlation between miR-122 levels and *Firmicutes* abundance (*r_s_* = 0.7160, *p* = 0.0027) (Table 3).

Meanwhile, Pearson’s analysis showed that levels of miR-370 exhibited a significant negative correlation with *Bacteroidetes* abundance (*r* = −0.8700, *p* = 0.0003) and a significant positive correlation with *Firmicutes* abundance (*r* = 0.7920, *p* = 0.0004). Spearman’s correlation analysis yields similar results, revealing a significant negative correlation between miR-370 and *Bacteroidetes* (*r_s_* = −0.8700, *p* < 0.0001) and a significant positive correlation between miR-370 and *Firmicutes* (*r_s_* = 0.8820, *p* < 0.0001) (Table 3).

Overall, the correlation results showed that, while the levels of miR-122 and miR-370 were significantly associated with the abundances of *Bacteroidetes* and *Firmicutes*, levels of miR-370 were more strongly associated with these abundances in the MetS and control groups. However, as our exploratory findings were based on a small sample size, the high |r| values may partly reflect the separation between the MetS and control groups rather than indicating a tight continuous relationship across all participants.

### 3.6. The Power of the Pilot Study

Between 2019 and 2021, the prevalence of MetS among 50-year-old Korean adults was reported to be 34.2% [45]. Using both the sample size calculator and the post hoc power calculator provided by ClinCalc (https://clincalc.com/), we calculated the power of our pilot study based on this information. The mean ages of the MetS and control groups enrolled in our pilot study were 56.1 and 54.3 years, respectively. Therefore, for a MetS prevalence of 34.2% in 50-year-old Koreans, with an alpha value of 0.05 and a beta value of 0.55, our study’s sample size (7 MetS and 8 controls) was found to have 45% power using the sample size calculator. In contrast, the post hoc power calculation revealed that our study had 43.5% power.

## 4. Discussion

In this pilot study, we demonstrated a close association between plasma levels of miR-122 and miR-370, which are related to lipid metabolism, and the abundances of the bacterial phyla *Firmicutes* and *Bacteroidetes* in individuals with MetS. Notably, there was a significant positive correlation between *Firmicutes* abundance and plasma levels of miR-122 and miR-370, whereas there was a significant negative correlation between *Bacteroidetes* abundance and each miR. These results suggest that these 2 miRs may act as biomarkers for predicting MetS. To the best of our knowledge, no previous study has reported significant correlations between *Bacteroidetes* and *Firmicutes* abundances at the phylum level and plasma levels of these 2 miRs.

Both GM and miRs are key factors in regulating human health, and recent studies have revealed their complex and bidirectional interactions in chronic diseases [33,46,47]. Conversely, the microbiota can influence the expression of miRs in the human body, thereby affecting inflammatory pathways and the onset of disease. Furthermore, miRs can influence gene expression and modulate the composition and function of GM, thereby significantly affecting metabolic and immune processes [48].

Dysbiosis is commonly observed in individuals with MetS [19,49]. As Figure 1 shows, our results revealed clear evidence of dysbiosis in the MetS group. The abundances of *Firmicutes* and *Bacteroidetes* were found to be 2.60 and 2.82 times higher and lower, respectively, in the MetS group than in the control group. The F/B ratio has been investigated as a potential biomarker for GM dysbiosis, with changes in this ratio being identified in MetS and related metabolic diseases. This suggests that the F/B ratio plays a crucial role in MetS and related metabolic diseases [22,50,51,52,53]. Consistent with previous studies, our results showed that the log F/B ratio, calculated using the declared CLR transformation, was significantly higher in the MetS group than in the control group. This indicates that an elevated log F/B ratio accompanied by dysbiosis, increases the likelihood of developing MetS and influences the onset of related metabolic diseases [22,50,51]. Furthermore, it may provide insights into the close relationships between the dominant phyla. Based on these findings, our pilot results suggest that the GM itself may be a potential therapeutic target for MetS. The GM abundance and the log F/B ratio at the phylum level may also serve as biomarkers for predicting MetS onset. However, as the GM abundance and the F/B ratio are known to vary by ethnicity [50], further studies are needed to evaluate potential therapeutic approaches using a large-scale Korean MetS cohort.

MiRs play a crucial role in maintaining intestinal epithelial cells. They are essential for absorbing necessary nutrients and regulating immune responses in the gut. This influences the balance between inflammation and immune tolerance [54]. Depending on an individual’s metabolic health characteristics, circulating miRs are also upregulated or downregulated. Both miR-15a-5p and miR-17-5p, which are known as biomarkers for predicting the onset of MetS, have been found to be downregulated in individuals with MetS [30,55,56]. Conversely, miR-21 is downregulated in MetS, but instead upregulated in patients with chronic heart disease and T2D [57]. A previous study reported that miR-370 regulates the expression of miR-122 in vitro, thereby affecting lipid metabolism [29]. Another study involving patients with hyperlipidemia found that levels of the 2 miRs related to lipid metabolism were significantly higher in the patient group than in the control group [44]. As hyperlipidemia is a risk factor for MetS, our study suggests that elevated plasma levels of miR-122 and miR-370 may be closely related to MetS in Koreans. Our results also showed that plasma levels of miR-122 and miR-370 were approximately 1.95- and 1.67-fold higher, respectively, in the MetS group than in the control group (Figure 2A,B). Notably, the AUC values for detecting MetS were 94.6% and 96.4% for miR-122 and miR-370, respectively, suggesting their potential as biomarkers for predicting MetS. However, given that the relatively small sample size of the study, the values are exploratory rather than definitive. Further study is therefore needed to validate these values. A recent study suggested that lipopolysaccharide and intestinal epithelial cell damage caused by gut dysbiosis may be associated with the upregulation of miR-122, which disturbs lipid metabolism. This suggests that the bidirectional interplay between GM and MetS may occur via inflammatory responses and changes in the gut bacterial composition [58]. While several studies have reported higher levels of miR-122 in the blood of individuals with MetS [58,59,60], the relationship between gut microbial composition at the phylum or genus level and plasma levels of miR-122 or miR-370 in individuals with MetS remains unclear.

As shown in Table 3, we demonstrated a significant positive correlation between the abundance of *Firmicutes* and the levels of both miR-122 and miR-370, as well as a significant negative correlation between the abundance of *Bacteroidetes* and the levels of both miRs. These results suggest that these miRs may play an important role in regulating human health through complex, bidirectional interactions in chronic metabolic diseases such as MetS [59].

The power of our study was found to be up to 45%. However, due to the small sample size and statistical power of our pilot study, our findings regarding the potential association between the GM and the 2 miRs are exploratory and hypothesis-generating, rather than definitive.

The limitations of our study include the relatively small sample size and the fact that we only analyzed the composition of gut microbes at the phylum level. Furthermore, we only investigated the associations between the GM and the two specific miRs related to lipid metabolism. Despite these limitations, we identified a clear association between GM composition and plasma levels of the two miRs. Therefore, a larger Korean MetS cohort study is needed to address these limitations.

## 5. Conclusions

Significant positive correlations were observed between elevated levels of plasma miR-122 and miR-370 and an increased abundance of the *Firmicutes* phylum in individuals with MetS. These exploratory findings could contribute to a better understanding of the pathophysiological roles of circulating miR-122 and miR-370, as well as the bidirectional interaction between the GM and the 2 miRs in MetS and related metabolic diseases. However, a large-scale cohort study of Korean individuals with MetS is required to elucidate the associations between the GM and the miRs in more detail at the phylum, genus, and species levels.

## Figures and Tables

**Table 1:** Clinical characteristics of study participants.

	Healthy Control	MetS	95% CI	*p* Value
Number of participants	8	7		
Age (years)	54.3 ± 13.7	56.1 ± 14.9	[−18.0, 15.0]	0.6431
Height (cm)	161.9 ± 7.7	169.3 ± 10.2	[−19.0, 4.7]	0.1824
Weight (kg)	57.1 ± 2.2	80.4 ± 12.1	[−34.5, −12.4]	0.0014
WC (cm)	75.6 ± 1.6	96.5 ± 6.9	[−27.0, −15.0]	0.0014
BMI (kg/m^2^)	21.9 ± 1.6	28.0 ± 2.9	[−9.4, −3.4]	0.0014
SBP (mmHg)	111.3 ± 10.2	126.0 ± 12.1	[−28.0, −5.0]	0.0319
DBP (mmHg)	67.7 ± 6.7	78.7 ± 5.5	[−19.0, −3.0]	0.0123
FBG (mg/dL)	91.2 ± 4.3	102.7 ± 5.0	[−17.0, −5.0]	0.0029
Total chol (mg/dL)	199.8 ± 13.2	232.9 ± 22.1	[−53.0, −13.0]	0.0128
HDL chol (mg/dL)	69.8 ± 11.1	50.4 ± 17.9	[−2.0, 36.0]	0.1043
LDL chol (mg/dL)	114.8 ± 16.3	143.0 ± 32.7	[−63.0, 7.0]	0.0559
TG (mg/dL)	96.6 ± 35.1	172.4 ± 80.0	[−161.0, 12.0]	0.0559
AST (U/L)	23.1 ± 5.7	31.4 ± 7.4	[−16.0, 0.0]	0.0479
ALT (U/L)	22.8 ± 7.1	38.7 ± 18.0	[−26.0, −3.0]	0.0199
γ-GTP (U/L)	17.2 ± 4.6	43.1 ± 19.8	[−43.0, −8.0]	0.0021
Serum Cr (mg/dL)	0.8 ± 0.1	0.8 ± 0.1	[−0.2, 0.1]	0.2998
eGFR (mL/min/1.73 m^2^)	88.6 ± 14.9	96.6 ± 13.1	[−28.0, 8.0]	0.2239
hs-CRP (mg/L)	0.5 ± 0.1	1.9 ± 2.0	[−1.7, −0.2]	0.0014
Insulin (µIU/mL)	3.2 ± 1.5	11.3 ± 3.1	[−9.5, −5.3]	0.0014
HOMA-IR	0.7 ± 0.3	2.8 ± 0.7	[−2.5, −1.5]	0.0013

The Wilcoxon–Mann–Whitney test was used to evaluate differences in clinical parameters between the MetS and control groups. Each value is shown as mean ± SD. CI, confidence interval; WC, waist circumference; BMI, body mass index; SBP, systolic blood pressure; DBP, diastolic blood pressure; FBG, fasting blood glucose; Total chol, total cholesterol; HDL chol, high-density lipoprotein-cholesterol; LDL chol, low-density lipoprotein cholesterol; TG, triglyceride; AST, aspartate aminotransferase; ALT, alanine aminotransferase; γ-GTP, γ-glutamyltranspeptidase; Serum Cr, serum creatinine; eGFR, estimated glomerular filtration rate; hs-CRP, high sensitivity-C-reactive protein; HOMA-IR, homeostatic model assessment of insulin resistance.

**Table 2:** Correlation analyses between the abundances of the *Bacteroidetes* and *Firmicutes* phyla and the clinical parameters of the study participants.

Clinical Parameters	*Bacteroidetes*						*Firmicutes*					
	Pearson			Spearman			Pearson			Spearman		
	*r* ^a^ [95% CI]	*p*	Adjusted *p* ^c^	*r_s_* ^b^ [95% CI]	*p*	Adjusted *p* ^c^	*r* ^a^ [95% CI]	*p*	Adjusted *p* ^c^	*r_s_* ^b^ [95% CI]	*p*	Adjusted *p* ^c^
WC	−0.7186 [−0.8997, −0.3265]	0.0025	0.0425	−0.6598 [−0.8786, −0.4502]	0.0074	0.1266	0.793 [0.4728, 0.9282]	0.0004	0.0068	0.6799 [0.4775, 0.9051]	0.0053	0.0900
BMI	−0.6775 [−0.8832, −0.2531]	0.0055	0.0468	−0.6897 [−0.8822, −0.4660]	0.0044	0.0377	0.7765 [0.4389, 0.9220]	0.0007	0.0040	0.7107 [0.4968, 0.8980]	0.003	0.0253
SBP	−0.5686 [−0.8371, −0.0795]	0.0270	0.0574	−0.6152 [−0.8909, −0.0074]	0.0146	0.0829	0.4909 [−0.0285, 0.8016]	0.0632	0.1194	0.6308 [0.0205, 0.8867]	0.0117	0.0662
DBP	−0.63 [−0.8636, −0.1738]	0.0118	0.0334	−0.746 [−0.9222, −0.3304]	0.0014	0.0060	0.5637 [0.0722, 0.8349]	0.0286	0.0608	0.6976 [0.2469, 0.9110]	0.0038	0.0163
FBG	−0.6692 [−0.8798, −0.2388]	0.0064	0.0363	−0.7178 [−0.9164, −0.4145]	0.0026	0.0088	0.7705 [0.4267, 0.9198]	0.0080	0.0227	0.6847 [0.3759, 0.8989]	0.0049	0.0165
Total chol	−0.6285 [−0.8629, −0.1714]	0.0121	0.0294	−0.7751 [−0.9312, −0.5568]	0.0007	0.0019	0.7884 [0.4633, 0.9265]	0.0005	0.0043	0.6893 [0.3480, 0.8909]	0.0045	0.0127
HDL chol	0.3309 [−0.2184, 0.7210]	0.2283	0.2985	0.2415 [−0.4484, 0.7674]	0.3859	0.9372	−0.6368 [−0.8664, −0.1848]	0.0107	0.0260	−0.2323 [−0.7623, 0.4507]	0.4048	0.9831
LDL chol	−0.4331 [−0.7737, 0.1018]	0.1068	0.1816	−0.5565 [−0.8462, 0.1413]	0.0312	0.0663	0.4752 [−0.0490, 0.7941]	0.0735	0.1136	0.4942 [−0.1557, 0.8117]	0.0612	0.1299
TG	−0.2376 [−0.6685, 0.3128]	0.3939	0.4185	−0.4651 [−0.8642, 0.2283]	0.0807	0.1524	0.4876 [−0.0328, 0.8000]	0.0652	0.1108	0.4458 [−0.2646, 0.8317]	0.0958	0.1809
AST	−0.3067 [−0.7078, 0.2439]	0.2662	0.3232	−0.3213 [−0.6938, 0.2379]	0.2429	0.4129	0.2505 [−0.3003, 0.6760]	0.3678	0.4168	0.3426 [−0.2034, 0.6989]	0.2112	0.3590
ALT	−0.2604 [−0.6817, 0.2907]	0.3487	0.3952	−0.3969 [−0.7503, 0.2210]	0.1429	0.2208	0.2374 [−0.3129, 0.6684]	0.3942	0.4188	0.4092 [−0.1976, 0.7670]	0.1299	0.2008
γ-GTP	−0.4084 [−0.7614, 0.1314]	0.1307	0.1852	−0.6962 [−0.9103, −0.4823]	0.0039	0.0056	0.4358 [−0.0984, 0.7750]	0.4358	0.4358	0.6643 [ 0.4345, 0.8846]	0.0069	0.0098
Serum Cr	−0.0058 [−0.5165, 0.5080]	0.9836	0.9836	−0.235 [−0.6592, 0.4125]	0.3992	0.5220	0.1737 [−0.3716, 0.6299]	0.1737	0.2271	0.247 [−0.3849, 0.6539]	0.3748	0.4901
eGFR	−0.4186 [−0.7665, 0.1192]	0.1204	0.1861	−0.526 [−0.7985, −0.0074]	0.044	0.0534	0.3184 [−0.2316, 0.7142]	0.2474	0.3004	0.5103 [0.0055, 0.8004]	0.052	0.0631
hs-CRP	−0.4825 [−0.7976, 0.0395]	0.0685	0.1294	−0.655 [−0.8668, −0.4162]	0.008	0.0091	0.4621 [−0.0657, 0.7879]	0.0829	0.1174	0.6625 [0.4237, 0.8853]	0.0071	0.0081
Insulin	−0.6433 [−0.8692, −0.1955]	0.0097	0.0330	−0.6971 [−0.8996, −0.4725]	0.0039	0.0041	0.6907 [0.2762, 0.8886]	0.0044	0.0150	0.7386 [0.5512, 0.9100]	0.0017	0.0018
HOMA-IR	−0.6635 [−0.8775, −0.2291]	0.007	0.0298	−0.7168 [−0.9028, −0.4894]	0.0026	0.0026	0.733 [0.3535, 0.9053]	0.0019	0.0081	0.746 [0.5553, 0.9167]	0.0014	0.0014

^a^ Pearson’s correlation coefficient. ^b^ Spearman’s correlation coefficient. ^c^ FDR-adjusted *p* value.

**Table 3:** Pearson’s and Spearman’s correlation analyses between the two phyla and the two miRs.

miRs	*Bacteroidetes*				*Firmicutes*			
	Pearson		Spearman		Pearson		Spearman	
	*r* ^a^ [95% CI]	*p*	*r_s_* ^b^ [95% CI]	*p* ^c^	*r* ^a^ [95% CI]	*p*	*r_s_* ^b^ [95% CI]	*p* ^c^
miR-122	−0.6890 [−0.8862, −0.2660]	0.0048	−0.6890 [−0.8988, −0.3542]	0.0045	0.6980 [0.2884, 0.8913]	0.0038	0.7160 [0.4007, 0.9235]	0.0027
miR-370	−0.8700 [−0.9307, −0.4870]	0.0003	−0.8700 [−0.9782, −0.7032]	<0.0001	0.7920 [0.4698, 0.9277]	0.0004	0.8820 [0.7308, 0.9845]	<0.0001

^a^ Pearson’s correlation coefficient, ^b^ Spearman’s correlation coefficient, ^c^ *p* value evaluated by 10,000 permutation tests.

## Data Availability

The original contributions presented in this study are included in the manuscript and Supplementary Materials. Additional inquiries may be directed to the corresponding authors.

## Supplementary Materials

The following supporting information can be downloaded at: https://www.mdpi.com/article/10.3390/genes16101161/s1, Table S1: Comparison of GM composition between MetS and control groups was analyzed using Mann–Whitney U test. Relative abundance values at the genus level are shown for the MetS and control groups. The *p* values and the FRD-adjusted *p* values were calculated respectively. Table S2: The relative expression levels of miR-122 and miR-370 in all study participants. Each value is expressed as mean ± SD.

## Abbreviations

The following abbreviations are used in this manuscript:

MetS	Metabolic syndrome
miR	MicroRNA
GM	Gut microbiota
F/B	*Firmicutes*/*Bacteroidetes*
CVD	*Cardiovascular disease*
T2D	Type 2 diabetes
ASV	Amplicon sequence variants
ROC	Receiver operating characteristic
AUC	Area under the curve
WC	Waist circumference
BMI	Body mass index
SBP	Systolic blood pressure
DBP	Diastolic blood pressure
FBG	Fasting blood glucose
Total chol	Total cholesterol
HDL chol	High-density lipoprotein cholesterol
LDL chol	Low-density lipoprotein cholesterol
TG	Triacylglyceride
AST	Aspartate aminotransferase
ALT	Alanine aminotransferase
γ-GTP	γ-glutamyltranspeptidase
Serum Cr	Serum creatinine
eGFR	Estimated glomerular filtration rate
Hs-CRP	High sensitivity-C-reactive protein
HOMA-IR	Homeostatic model assessment of insulin resistance
NGS	Next-generation sequencing
RT-qPCR	Real-time quantitative PCR
