# Supplementary material for: Significant Association Between Abundance of Gut Microbiota and Plasma Levels of microRNAs in Individuals with Metabolic Syndrome and Their Potential as Biomarkers for Metabolic Syndrome: A Pilot Study
Source: Genes (Basel). 2025 Sep 30;16(10):1161. doi: 10.3390/genes16101161 (PMC12563822; doi:10.3390/genes16101161)
Supplement: Supplementary file 1 [file genes-16-01161-s001.zip › genes-3870871-supplementary.pdf]

Supplementary Table S1. Comparison of GM composition between MetS and control groups was analyzed using Mann-Whitney U test. Relative abundance values at the genus level are shown for the MetS and control groups. The *p* values and the FRD-adjusted *p* values were calculated, respectively.

Genus	MetS group							Healthy control group								*p* value	FDR-adjusted *p* value
	Subject 1	Subject 2	Subject 3	Subject 4	Subject 5	Subject 6	Subject 7	Subject 1	Subject 2	Subject 3	Subject 4	Subject 5	Subject 6	Subject 7	Subject 8		
Catenibacterium	0.035667	0.000223	0	0.002993	0	0.001819	0	0	0	0	0	0	0	0	0	0.010664963	1.000
Intestinibacter	0.005262	0	0.000246	0.00288	0	0.001054	0	0	0	0	0	0	0	0	0	0.010664963	0.619
Holdemanella	0.003313	0	0	0.006213	0	0.002718	0	0	0	0	0	0	0	0	0	0.02854475	1.000
Intestinimonas	0	0	0.00258	0.007737	0	0.000255	0	0	0	0	0	0	0	0	0	0.02854475	0.828
Negativibacillus	0	0	0.000404	0.001243	0	0.000416	0	0	0	0	0	0	0	0	0	0.02854475	0.662
Romboutsia	0	0	0.000955	0.006382	0	0.000685	0	0	0	0	0	0	0	0	0	0.02854475	0.552
Collinsella	0.004873	0.003586	0.012485	0.010731	0	0.001027	0	0	0.0012876	0	0	0	0	0.0085164	0	0.045077989	0.747
Klebsiella	0	0	0	0	0	0	0	0.0039	0	0	0	0	0.3105201	0	0.000444	0.049017398	0.711
Veillonella	0.007699	0.006094	0	0	0.013393	0.000503	0.005343	0	0	0.00818553	0	0	0	0.0040965	0	0.058317982	0.752
Coprobacter	0	0	0.003466	0	0	0.002282	0	0	0	0	0	0	0	0	0	0.071172524	0.826
Bilophila	0	0	0.000305	0.004518	0	0	0	0	0	0	0	0	0	0	0	0.071172524	0.751
Candidatus Soleaferrea	0	0	0	0.000678	0	0.000114	0	0	0	0	0	0	0	0	0	0.071172524	0.688
Family XIII AD3011 group	0	0	0	0.001525	0	0.000564	0	0	0	0	0	0	0	0	0	0.071172524	0.635
Mitsuokella	0.001511	0	0	0	0	0.002859	0	0	0	0	0	0	0	0	0	0.071172524	0.590
Dialister	0	0	0	0.042697	0	0.175146	0	0	0	0	0	0	0	0	0	0.071172524	0.550
Haemophilus	0.030307	0.001254	0	0	0	0	0	0	0	0	0	0	0	0	0	0.071172524	0.516
Faecalibacterium	0.064464	0.043583	0.031133	0.170677	0.100236	0.027452	0.038187	0.4475053	0.0854619	0.01152749	0.3067894	0.78479254	0.17340426	0.035618	0.11625412	0.074005347	0.505
Monoglobus	0.004629	0.000461	0.004549	0.004631	0	0.003994	0	0.0027238	0	0	0	0	0.00526005	0	0	0.074435997	0.480
Coprococcus	0	0	0	0.020502	0	0.00196	0	0.0381949	0.0041244	0	0.0271577	0.00264708	0.01317967	0	0	0.08367558	0.511
Colidextribacter	0	0	0.000276	0.000791	0	0.00092	0	0	0.000492	0	0	0	0	0	0	0.10339559	0.600
Prevotella_9	0.454417	0.786306	0.001014	0.007737	0	0	0.762261	0.0909372	0	0	0.3380898	0	0	0.4733188	0	0.123252279	0.681
Butyricicoccus	0	0	0	0.015983	0	0.000557	0	0.0029714	0.0052759	0	0.0029919	0.00787506	0.00437352	0	0	0.129297745	0.682
Blautia	0.020416	0.000306	0.001694	0.005535	0	0.00153	0	0	0.0263378	0	0	0.00840447	0	0	0	0.142996091	0.721
Alloprevotella	0.007796	0.032096	0	0	0	0	0.037888	0.0053238	0	0	0	0.00297796	0	0	0	0.150694521	0.728
Ruminococcus	0.008819	0.001145	0.032088	0.031515	0	0.007893	0	0.0229045	0	0.00451999	0.0048331	0	0.00827423	0	0	0.154827952	0.718
Alistipes	0	1.04E-05	0.004421	0.002485	0	0.011873	0	0.0033428	0.07111	0	0.0204833	0.00291179	0.00183215	0.005153	0	0.158235894	0.706
Megamonas	0.02943	0.001881	0	0.108494	0	0	0.037788	0.0503281	0	0	0	0.02944875	0	0	0	0.163743841	0.703
Anaerostipes	0.01184	0.000705	0	0	0	0.000718	0	0	0.025626	0	0	0	0	0	0	0.167173789	0.693
[Eubacterium] eligens group	0.00536	0	0.000502	0.003671	0	0	0	0.0263712	0.018382	0	0.0174914	0	0.00596927	0	0	0.173242059	0.693
Adlercreutzia	0	0	0	0	0	0.000765	0	0	0	0	0	0	0	0	0	0.174787403	0.676
Slackia	0	0	0	0.005535	0	0	0	0	0	0	0	0	0	0	0	0.174787403	0.654
Sanguibacteroides	0	0	0.000384	0	0	0	0	0	0	0	0	0	0	0	0	0.174787403	0.634
Prevotellaceae UCG-003	0	0	0	0.00288	0	0	0	0	0	0	0	0	0	0	0	0.174787403	0.614
Desulfovibrio	0	0	0	0.012199	0	0	0	0	0	0	0	0	0	0	0	0.174787403	0.596
[Clostridium] innocuum group	0	0	0.000276	0	0	0	0	0	0	0	0	0	0	0	0	0.174787403	0.579
Eubacterium	0	0	0.000945	0	0	0	0	0	0	0	0	0	0	0	0	0.174787403	0.563
Defluviitaleaceae UCG-011	0	0	0	0	0	0.000329	0	0	0	0	0	0	0	0	0	0.174787403	0.548
CAG-56	0	0	0	0	0	0.002859	0	0	0	0	0	0	0	0	0	0.174787403	0.534
Eisenbergiella	0	0	0	0	0	0.00149	0	0	0	0	0	0	0	0	0	0.174787403	0.520
GCA-900066575	0	0	0	0.00209	0	0	0	0	0	0	0	0	0	0	0	0.174787403	0.507
Hungatella	0	0	0	0	0	0.00196	0	0	0	0	0	0	0	0	0	0.174787403	0.495
Tyzzerella	0	0	0	0.00209	0	0	0	0	0	0	0	0	0	0	0	0.174787403	0.483

UCG-009	0	0	0	0	0	0.000235	0	0	0	0	0	0	0	0	0	0.174787403	0.472
Acetanaerobacterium	0	0	0	0	0	6.71E-05	0	0	0	0	0	0	0	0	0	0.174787403	0.461
Ruminiclostridium	0	0	0	0	0	0.00096	0	0	0	0	0	0	0	0	0	0.174787403	0.451
Pseudoflavonifractor	0	0	0.001083	0	0	0	0	0	0	0	0	0	0	0	0	0.174787403	0.441
Anaerotruncus	0	0	0	0	0	0.000322	0	0	0	0	0	0	0	0	0	0.174787403	0.431
Angelakisella	0	0	0	0	0	5.37E-05	0	0	0	0	0	0	0	0	0	0.174787403	0.422
Caproiciproducens	0	0	0	0.000452	0	0	0	0	0	0	0	0	0	0	0	0.174787403	0.414
DTU089	0	0	0	0	0	0.000295	0	0	0	0	0	0	0	0	0	0.174787403	0.406
Paludicola	0	0	0	0	0	0.000799	0	0	0	0	0	0	0	0	0	0.174787403	0.398
Allisonella	0	0	0	0	0	0.002906	0	0	0	0	0	0	0	0	0	0.174787403	0.390
Megasphaera	0	0	0	0	0	0.0574	0	0	0	0	0	0	0	0	0	0.174787403	0.383
Fusobacterium	0	0	0	0.012256	0	0	0	0	0	0	0	0	0	0	0	0.174787403	0.375
Citrobacter	0	0	0.000118	0	0	0	0	0	0	0	0	0	0	0	0	0.174787403	0.369
Raoultella	0	0.000135	0	0	0	0	0	0	0	0	0	0	0	0	0	0.174787403	0.362
Succinatimonas	0.000585	0	0	0	0	0	0	0	0	0	0	0	0	0	0	0.174787403	0.356
Parabacteroides	0.001169	0	0.016354	0.018751	0.032434	0.000973	0	0.0100904	0.0638975	0	0	0	0.00874705	0	0	0.180120406	0.360
[Ruminococcus] torques group	0.002582	0	0.000236	0.014797	0	0.005215	0	0	0.0123629	0	0	0	0.00945627	0	0	0.198044001	0.389
Lachnospira	0.011256	0.002073	0	0.013555	0	0.001094	0	0.0070571	0	0	0	0	0.02399527	0	0	0.198044001	0.383
Agathobacter	0.022365	0.001378	0	0.019428	0	0.003443	0	0.0689612	0.007223	0.00651202	0	0.03480908	0.0358747	0	0	0.201391847	0.383
Bifidobacterium	0	0.001897	0	0	0	0.011202	0	0	0.0013818	0	0	0	0	0	0	0.204053853	0.382
Lachnospiraceae UCG-010	0	0	0	0.004292	0	0	0	0.0037143	0	0	0	0.0043015	0.00336879	0	0	0.207000869	0.381
Eggerthella	0	0	0	0	0	0	0	0	0	0.00038425	0	0	0	0	0	0.211339037	0.383
Erysipelatoclostridium	0	0	0	0	0	0	0	0.0009905	0	0	0	0	0	0	0	0.211339037	0.377
Lactococcus	0	0	0	0	0	0	0	0.0005571	0	0	0	0	0	0	0	0.211339037	0.371
[Ruminococcus] gauvreauii group	0	0	0	0	0	0	0	0	0.0055795	0	0	0	0	0	0	0.211339037	0.366
Lachnospiraceae UCG-001	0	0	0	0	0	0	0	0	0	0	0	0	0.00130024	0	0	0.211339037	0.361
Lachnospiraceae UCG-003	0	0	0	0	0	0	0	0	0	0	0	0	0.00921986	0	0	0.211339037	0.355
Tuzzerella	0	0	0	0	0	0	0	0	0.0024809	0	0	0	0	0	0	0.211339037	0.350
Anaerovibrio	0	0	0	0	0	0	0	0.0074285	0	0	0	0	0	0	0	0.211339037	0.345
Parasutterella	0	0	0	0	0	0	0	0	0	0	0	0	0.00543735	0	0	0.211339037	0.340
[Eubacterium] ruminantium group	0.01452	0	0	0.005591	0	0.000819	0	0.0039619	0	0	0	0	0.00242317	0	0	0.224264376	0.356
NK4A214 group	0	0	0.00064	0.016492	0	0.003631	0	0.0027857	0	0	0	0	0.00212766	0	0	0.224264376	0.352
Barnesiella	0	0	0.016965	0	0	0	0	0.0022905	0	0	0	0	0.00218676	0	0.01550302	0.251955232	0.390
Butyricimonas	0	0	0.000601	0.002542	0	0	0	0	0	0	0	0	0	0.0017895	0	0.254055281	0.388
Turicibacter	0.002534	0	7.88E-05	0	0	0	0	0	0	0	0	0	0.00183215	0	0	0.254055281	0.383
UCG-003	0.002875	0	0	0	0	0.000664	0	0	0	0	0	0	0.00171395	0	0	0.254055281	0.378
Akkermansia	0	0	0	0.026488	0	0.111054	0	0	0	0	0	0	0	0	0.05464906	0.254055281	0.373
Lachnospiraceae NK4A136 group	0.015495	0.001788	0.001319	0.002542	0	0.002322	0	0.0097809	0.002973	0	0.0140391	0.01191185	0.02588653	0	0	0.258433875	0.375
Prevotella	0.042343	0.048491	0	0	0	0.00049	0.032209	0.0033428	0	0	0	0.00357356	0	0.1564218	0	0.26512627	0.380
Sutterella	0.036057	0.00227	0.003919	0.012877	0	0	0	0.0124427	0.0101541	0	0	0	0.00644208	0	0	0.26512627	0.375
Paraprevotella	0.007017	0.006519	0	0.001694	0	0	0.006114	0	0	0	0	0	0	0.0184127	0.07588708	0.278429917	0.389
Erysipelotrichaceae UCG-003	0	0	0.002107	0	0	0	0	0	0.0072021	0	0	0	0.00449173	0	0	0.281274422	0.388
Prevotellaceae Ga6A1 group	0	0	0.051475	0.017621	0	0	0	0	0	0	0	0	0	0	0.05315055	0.309828352	0.423
Fusicatenibacter	0	0	0.000315	0	0	0.001852	0	0	0	0	0	0.00357356	0	0	0	0.309828352	0.418
Christensenellaceae R-7 group	0	0	0.001566	0.002654	0	0.015061	0	0.0029095	0	0	0	0	0.00679669	0	0	0.314803181	0.420
Incertae Sedis	0.000292	0	0	0.000791	0	0.001644	0	0.0006809	0.001476	0	0	0.0057574	0.00118203	0	0	0.33020963	0.435
Roseburia	0	0	0	0.010561	0	0.004893	0	0.0104618	0	0	0.0094361	0	0.14154846	0	0	0.339662761	0.443
Subdoligranulum	0.007553	0.002658	0.001408	0.012821	0	0.143533	0	0.0178903	0.0036115	0	0.0197929	0.04327973	0.02996454	0	0.000555	0.341375533	0.440

UCG-002	0.003801	0	0.001211	0.014458	0	0.055782	0	0.0137427	0.0107822	0	0.0197929	0.01475746	0.01093381	0	0	0.359958927	0.459
Lachnospiraceae ND3007 group	0.00268	0	7.88E-05	0	0	0.001195	0	0	0.0060506	0	0	0	0.00218676	0	0	0.365240258	0.461
Lachnoclostridium	0.001511	0	4.92E-05	0.018864	0	0.001792	0	0.0151665	0	0	0.010817	0.00463239	0.02310875	0	0	0.380194968	0.474
Phascolarctobacterium	0.038298	0.018411	0.081131	0.043432	0	0	0.031302	0.0532995	0	0	0.0547756	0.00900007	0.02553192	0.0688644	0.05768306	0.385167357	0.475
Dorea	0.007406	0	0	0	0	0.00094	0	0.0023524	0.0057261	0	0	0	0.00189125	0	0	0.391431831	0.478
UCG-005	0	0	0	0.003106	0	0.024391	0	0	0.0035696	0	0	0	0.00242317	0	0	0.411852123	0.498
Odoribacter	0	0	0.008458	0	0.019528	0.000597	0	0.0008048	0.0160267	0	0	0	0	0	0.00688201	0.422368732	0.505
Bacteroides	0.026897	0.025219	0.653795	0.044448	0.83285	0.060749	0.030595	0.0139284	0.5029101	0.96795053	0.1360184	0.01766925	0.04131206	0.1320153	0.57448107	0.431093347	0.510
[Eubacterium] hallii group	0	0	0	0	0	8.05E-05	0	0	0.0058831	0	0	0	0	0	0	0.461042536	0.540
[Eubacterium] xylanophilum group	0	0	0	0	0	0.001618	0	0	0	0	0	0	0.00667849	0	0	0.461042536	0.535
[Ruminococcus] gnavus group	0	0	0.000177	0	0	0	0	0	0.0216376	0	0	0	0	0	0	0.461042536	0.530
Lachnospiraceae FCS020 group	0	0	0	0	0	0.000309	0	0	0	0	0	0	0.0035461	0	0	0.461042536	0.524
Lachnospiraceae UCG-004	0.001949	0	0	0	0	0	0	0	0	0	0	0	0.00738771	0	0	0.461042536	0.519
Flavonifractor	0	0	0	0	0	0.000436	0	0	0.0006909	0	0	0	0	0	0	0.461042536	0.514
UBA1819	0	0	0	0	0	0.00194	0	0	0.0033393	0	0	0	0	0	0	0.461042536	0.509
Family XIII UCG-001	0	0	0	0	0	0.000631	0	0.0004952	0	0	0	0	0	0	0	0.461042536	0.505
TM7x	0	0	0	0	0	0.000195	0	0	0	0	0	0.00191913	0	0	0	0.461042536	0.500
Enterobacter	0.005019	0	0	0	0	0	0	0	0	0.00010112	0	0	0	0	0	0.461042536	0.495
Escherichia-Shigella	0	0	0	0	0	0.00494	0	0	0.0029311	0	0	0	0	0	0	0.461042536	0.491
Succinivibrio	0	0	0	0.005874	0	0	0	0.0025381	0	0	0	0	0	0	0	0.461042536	0.486
Streptococcus	0.001559	0	0	0	0	0.001222	0	0	0.0027112	0	0	0	0.00200946	0	0	0.470401328	0.492
[Eubacterium] ventriosum group	0	0	0	0.001017	0	0.000933	0	0.0071809	0	0	0	0	0.00159575	0	0	0.470401328	0.487
[Eubacterium] siraeum group	0	0	0	0.004123	0	7.38E-05	0	0	0.0135667	0	0	0	0.00271868	0	0	0.470401328	0.483
CAG-352	0	0	0	0.006043	0	0.016162	0	0	0.0077988	0	0.0128884	0	0	0	0	0.470401328	0.479
Clostridium sensu stricto 1	0.003362	9.85E-05	0.001014	0	0	0	0	0.0017333	0.0021983	0	0	0.00185296	0	0	0	0.473975883	0.478
Oscillibacter	0	0	0.000926	0.002824	0	0.000658	0	0.0013	0.0091387	0	0	0	0.00076832	0	0	0.473975883	0.474

Supplementary Table S2. The relative expression levels of miR-122 and miR-370 in all study participants. Each value is expressed as mean±SD.

miRs	Healthy controls								MetS							p value
	**H1**	**H2**	**H3**	**H4**	**H5**	**H6**	**H7**	**H8**	**M1**	**M2**	**M3**	**M4**	**M5**	**M6**	**M7**	
miR-122	1.14±0.00	1.08±0.01	0.41±0.05	0.47±0.07	0.95±0.07	0.62±0.00	0.69±0.01	0.51±0.01	1.39±0.00	1.45±0.01	1.60±0.05	0.68±0.08	0.94±0.53	1.99±0.21	1.96±0.09	0.0065
miR-370	0.22±0.18	1.32±0.06	0.68±0.08	0.83±0.72	1.06±0.23	1.04±0.01	0.84±0.01	0.65±0.01	1.07±0.00	1.58±0.00	1.62±0.01	1.14±0.44	1.55±0.11	1.28±0.2	1.57±0.17	0.0089
